# Variation in Recorded Child Maltreatment Concerns in UK Primary Care Records: A Cohort Study Using The Health Improvement Network (THIN) Database

**DOI:** 10.1371/journal.pone.0049808

**Published:** 2012-11-28

**Authors:** Jenny Woodman, Nick Freemantle, Janice Allister, Simon de Lusignan, Ruth Gilbert, Irene Petersen

**Affiliations:** 1 MRC (Medical Research Council)-Centre of Epidemiology for Child Health, University College London (UCL)-Institute of Child Health, London, United Kingdom; 2 University College London (UCL)-Department of Primary Care and Population Health, University College London (UCL), London, United Kingdom; 3 Royal College of General Practitioners (RCGP), London, United Kingdom; 4 Department of Health Care Management and Policy, University of Surrey, Guilford, United Kingdom; Indiana University and Moi University, United States of America

## Abstract

**Objectives:**

To determine variation over time and between practices in recording of concerns related to abuse and neglect (maltreatment) in children's primary care records.

**Design:**

Retrospective cohort study using a United Kingdom representative primary care database.

**Setting:**

448 General Practices.

**Participants:**

In total 1,548, 972 children (<18 y) registered between 1995 and 2010.

**Main Outcome Measures:**

Change in annual incidence of one or more maltreatment-related codes per child year of registration. Variation between general practices measured as the proportion of registered children with one or more maltreatment-related codes during 3 years (2008–2010).

**Results:**

From 1995–2010, annual incidence rates of any coded maltreatment-related concerns rose by 10.8% each year (95% confidence interval 10.5, 11.2; adjusted for sex, age and deprivation). In 2010 the rate was 9.5 per 1000 child years (95%CI: 9.3, 9.8), equivalent to a prevalence of 0.8% of all registered children in 2010. Across all practices, the median prevalence of children with any maltreatment-related codes in three years (2008 to 2010) was 0.9% (range 0%–13.4%; 11 practices (2.5%) had zero children with relevant codes in the same period). Once we accounted for sex, age, and deprivation, the prevalence for each practice was within two standard errors of the grand mean.

**Conclusions:**

General Practitioners (GPs) are far from disengaged from safeguarding children; they are consistently and increasingly recording maltreatment concerns. As these results are likely to underestimate the burden of maltreatment known to primary care, there is much scope for increasing recording in primary care records with implications for resources to respond to concerns about maltreatment. Interventions and policies should build on this evidence that the average GP in the UK is engaged in child safeguarding activity.

## Introduction

Child maltreatment (abuse and neglect) is common in high income countries: large, population-based studies using self/parent-reports estimate that 4% of children in the United Kingdom and 10% in the USA experience maltreatment each year [1], [2], [3]. Increasingly it is being recognised that health professionals have a role to play for maltreated children, particularly where concerns do not meet the threshold for child protection or social care services [4], [5], [6].

Due to regular contact with families and relationships with multiple family members over long periods of time, General Practitioners (GPs) have been seen as central to identifying and responding to children who are maltreated or are at risk of being maltreated [7], [8], [9]. However, there are doubts about how far this potential is fulfilled in practice. In qualitative studies, GPs and other stakeholders question whether the average GP have the expertise, time or inclination to recognise and respond to child maltreatment [10]. Social workers and health visitors describe GPs acting on the periphery of child protection [11]. Studies which report (historic) low GP attendance at child protection conferences seems to support this lack of GP engagement [12]. It is difficult to generalise from qualitative studies and there is little large-scale population-based evidence about how often GPs recognise and respond to maltreatment concerns in children and how these responses vary according to child characteristics and by practice.

One way we can begin to answer basic questions about frequency and patterns of responses to maltreatment identified in general practice is to analyse recorded concerns in the primary care record. The National Institute for Health and Clinical Excellence (NICE) and the General Medical Council (GMC) recommend that health professionals record concerns, including any “minor concerns” [4], [5]. Recording concerns can aid decision-making and patient management by building up a cumulative picture over time as well as facilitating information sharing; it is a necessary part of responding to maltreatment. We used codes in the primary care record to estimate the frequency of GP responses to child maltreatment.

## Methods

### Ethics

The THIN scheme for obtaining and providing anonymous patient data to researchers was approved by the National Health Service South-East Multicentre Research Ethics Committee in 2002 and scientific approval for this study using THIN was obtained from the Medical Research Scientific Review Committee in May 2011.

### Aim

We aimed to determine variation over time and between practices in recorded child maltreatment by using maltreatment-related codes in children's primary care records.

### Data source

Approximately 98% of the population in the UK is registered with a GP [13]. The Health Improvement Network (THIN) primary care database is one of the largest national collections of primary care data; in 2010 THIN contained data on over 10 million patients and covered 6% of the UK primary care population [14]. THIN is broadly representative of the GP population in terms of patient demographics, prevalence of codes for major conditions (e.g. asthma, stroke, diabetes) and mortality [15]. THIN can be considered representative of the UK population in terms of age and sex distribution, though there are slightly fewer patients aged under 15 compared to the general population and the male population matches slightly less well than the female [15]. Although practices which choose to contribute data may not be completely representative of all practices in the UK, the impact of any bias is unclear and is likely to be minimal as the dataset includes a substantial proportion of patients and practitioners in the UK. Diseases, symptoms, patient characteristics and problems are coded by primary care staff including GPs, nurses and administrative staff, currently using the Read version 2 system [16]. THIN makes available the 5-byte code plus the 2 byte term code. Diagnoses recorded by Read codes have moderate accuracy compared with reference clinical datasets for a range of conditions [17]. For each registered patient, deprivation is available in THIN in the form of quintiles of Townsend score, which is a composite measure of social deprivation based on postcode of residence linked to census data [18].

### Study population

We included children aged up to 18 years who were permanently registered with any of 448 participating General Practices at any point between January 1995 and December 2010. Time at risk started at the latest of: 1^st^ January 1995; child's registration; or the date when the practice met criteria for acceptable quality of mortality recording [19]. To avoid misclassifying prevalent cases as incident, we excluded the first five months of time at risk following registration (with the exception of children who were aged under one year at the time of registration, for whom this was likely to have been the first registration at a GP practice) [20]. Time at risk ended at the earliest of: 31^st^ December 2010; child's 18^th^ birthday; child's transfer out of the practice; child's date of death; or when the practice stopped contributing data.

### Identifying children with maltreatment-related codes

A wide variety of codes may indicate concerns about maltreatment in routine healthcare records, including indirect or euphemistic codes [21], [22]. We developed a Read code list to identify ‘maltreatment-related’ codes, which was designed to capture clinical concern about possible, probable or confirmed maltreatment; recognising that this clinical concept may be represented in many different parts of the coding system [23]. We used codes which ranged from the specific to the more sensitive (Table 1). Our main analyses were based on any maltreatment-related code in the child's primary care record, with sub-analyses based on the four sub-categories of codes in Table 1. Free text entries, which are idiosyncratic and difficult to collate on a population basis, were not available for this study.

**Table 1:** List of ‘maltreatment-related’ codes used to ascertain cases in THIN (Read codes shown are codes used ≥50 times in our dataset in 2009–10, ordered by code chapter).

Category (N of codes in category)	Example Read codes*
**1. Child protection procedures (N = 25)**	13IC. At risk register
Codes indicating child protection plan, case conference, or child protection investigation. Child protection plans are UK statutory child protection services, which can be roughly equated with ‘substantiated’ cases of maltreatment in North America and Australia [37].	13IM. Child on protection register
	13Id. On child protection register
	13Iv. Subject to child protection plan
	Z35.. Child protection procedure
	3874. Multidisciplinary case conference
	3875. Social services case conference
	3879. Review case conference
	64c.. Child protection procedure
	8CM6. Child protection plan
	9F2.. Child at risk-case conference
	Z331. Child protection plan
	Z352. Child protection investigation
**2. Direct references to maltreatment or out-of-home-care (N = 129)**	13VF. At risk violence in the home
Codes making explicit reference to abuse or neglect (including domestic violence) or to formal out-of-home care. In 2010–11 over 85% of Looked After children were in out-of-home care due to abuse or neglect or social problems in the family [25].	13IB. Child in care
	13IB0 Child in foster care
	13IV. Looked after child - Children (Scotland) Act 1995
	13ZV. At risk of neglect by others
	13ZT. At risk of physical abuse
	13HP6 Violence between parents
	13ZR. At risk of emotional/psychological abuse
	38C0. Child in care health assessment
	6982. Fostering medical examination
**3. High risk child (N = 131)**	13If. Child is cause for concern
Codes indicating high levels of social welfare need or concern in the child or family, including a history of abuse or neglect.	13Ip. Family is cause for concern
	13IF. Child at risk
	13IF.11 Vulnerable child**
	13IQ. Vulnerable child in family
	13IS. Child in need
	14XD. History of domestic abuse
	14X3. History of domestic violence
	13W..11 Family problems
	1BE1. Problem situation
	625.. A/N care: social risk
	8CM5. Child in need plan
**4. Contact with children's social care (N = 66)**	13G4. Social worker involved
Codes indicating that the child is involved with or has been referred to children's social care (not including codes specifically referring to ‘child protection procedure’ codes).	64RA.11 Child referral-social services
	8H75. Refer to social worker
	8HHB. Referral to Social Services
	9NDA. Report received from social services
	9N26. Seen by social worker
	9NNV. Under care of social services
	9Nl6. Seen by social services
	9b0k. Social services report

* Read Version 2 (5-Byte).For more detail on frequency of codes in THIN, sees ‘supplementary material’ at www.clininf.eu/maltreatment.

** 13IF code with a more specific meaning due to the addition of the “.11” term code.

The method for deriving the list of maltreatment-related codes was based on an audit of 11 practices conducted with the RCGP, reported elsewhere [24]. In brief, codes were selected to reflect a threshold that should trigger further action by health professionals, thereby meeting the threshold for ‘consider’ maltreatment as described in the 2009 NICE guidance [4]. A validation exercise of these codes in three practices indicated high specificity for ‘considered’ maltreatment [24].

### Socio-demographic characteristics

We adjusted analyses for age, gender and deprivation (quintile of Townsend score) as these factors are known confounders for variation in maltreatment. We used five developmental age groups, <1 y, 1–4 y, 5–9 y, 10–15 y, 16–<18 y, which were also comparable with national data from the Department of Education [25].

We restricted analyses to children with complete age and sex data. Missing data for the Townsend score were included in all analyses as an extra category of the deprivation variable. See Table 2 for more details.

**Table 2:** Characteristics of children included in the cohort.

	1995–2010			1998–2010		
	448 practices contributed data			443 practices contributed data		
	≥1 maltreatment-related code	All children	Years at risk	≥1 maltreatment-related code	All children	Years at risk
	N (%)		Sum (median)	N (%)		Sum (median)
**All children**	33,191 (2.1)	1,548,972 (100)	7,460,888.6 (3.9)	14,441 (1.5)	955,267 (100.0)	2,068,974.4 (2.8)
Boy	16,169 (48.7)	800,141 (51.7)	3,868,999 (3.9)	7,077 (49.0)	489,361 (51.2)	1.060,032.1 (2.8)
Girl	17,022 (51.3)	748,831 (48.3)	3,591,890 (3.8)	7,364 (51.0)	465,906 (48.8)	1,008,942.3 (2.8)
<1 y*	13,591 (40.9)	496,049 (32.0)	2, 296,494 (3.5)	3, 853 (26.7)	170,290 (17.8)	271,237 (1.5)
1–4 y*	7,125 (21.5)	290,091 (18.7)	1, 798,649 (5.5)	3,595 (24.9)	189,036 (19.8)	445,507 (3.0)
5–9 y*	6,925 (20.9)	319,429 (20.6)	1, 985,803 (6.9)	3,123 (21.6)	218,014 (22.8)	550,581 (3.0)
10–15 y*	5, 049 (15.2)	334,221 (21.6)	1, 283,161 (3.5)	3,447 (23.9)	282,827 (29.6)	713,482 (3.0)
16–17 y*	501 (1.5)	109,182 (7.0)	96, 747 (0.7)	423 (2.9)	95,100 (10.0)	88,167 (0.5)
Least deprived†	3,620 (10.9)	363,277 (23.5)	1,969,740 (4.7)	1,470 (10.5)	228,025 (24.6)	523,542 (3.0)
2†	3,607 (10.9)	301,922 (19.5)	1,5202,54 (4.2)	1,531 (11.0)	187,823 (20.2)	418,232 (3.0)
3†	6,254 (18.8)	310,661 (20.1)	1,478,873 (3.8)	2,704 (19.4)	192,103 (20.7)	412,788 (2.7)
4†	9,282 (28.0)	304,551 (19.7)	1,373,370 (3.5)	3,964 (28.4)	183,669 (19.8)	382,596 (2.5)
Most	9,678 (29.2)	231,473 (14.9)	1,006,997 (3.3)	4,283 (30.7)	183,334 (14.7)	282,003 (2.5)
Missing†	750 (2.3)	370,96 (2.4)	111,656 (2.1)	489 (3.4)	27,313 (2.4)	49,823 (1.9)

### Statistical analyses

#### Variation over time

To examine variation in maltreatment-related codes in the time period between 1995 and 2010, we calculated change in annual incidence and 95% confidence intervals (CI) for any maltreatment-related code and for the four sub-categories (Table 1). We divided the annual number of incident cases (first case per year per child) by the total child years at risk for each calendar year. We calculated rate ratios for sex and age and deprivation categories for annual incidence in 2010.

We calculated prevalence estimates for 2010 in order to judge whether rates for infants were inflated due to delays in GP registration following birth, which could shorten the denominator without necessarily reducing the numerator. Prevalence estimates also facilitated comparison of our results with results from existing literature and national data. We calculated prevalence by dividing the total number of children with a code in 2010 by the total number of children registered at any point in 2010.

We fitted negative binomial regression models for a log linear trend with a random intercept for practice, adjusted for sex, age category, and deprivation quintile. The negative binomial model was chosen because of evidence of over-dispersion (log likelihood ratio test (p<0.0001)). We based our model selection strategy on Akaike's information criterion (AIC) and took a pre-defined difference of −9 (three standard errors) to indicate a substantially better model fit [26]. The log linear model was selected over a basic linear model and a linear model with a 2005 change point (2005 was the first full calendar year with pay for performance (P4P) coding for some conditions in the UK following the introduction of the Quality and Outcomes Framework (QOF)) [27]. Because the univariate analyses showed greater difference between deprivation quintiles in young compared with older children, we included an interaction term between age and deprivation, which substantially improved the model fit.

To test whether our definition of population or time at risk substantially affected the results, we conducted sensitivity analyses for incidence rates in 2010, including temporarily registered patients and with and without including the first five months of time at risk following registration for all children.

#### Variation by practice

We grouped data for the three most recent years to examine current variation by practice (2008–2010). As we were interested in variation (not absolute measures), we used a logistic regression model to compare the prevalence of any maltreatment-related code between practices, adjusting for sex, age-category and deprivation quintile. We calculated the number of standard errors between the mean prevalence for each practice and the grand mean (mean of all 443 practices). We pre-defined outlying values as those more than three standard errors (SEs) above or below the grand mean. In the event of outliers, we planned to adjust for over-dispersion to avoid false positive identification of outliers.

All analyses were conducted in Stata, version 11.2 (Stata Corp, College Station, Texas) or SAS, version 9.2 (SAS Institute Inc, Cary, NC)

## Results

Table 2 reports the number of contributing practices and characteristics of our cohorts.

### Variation over time

**Figure 1:**
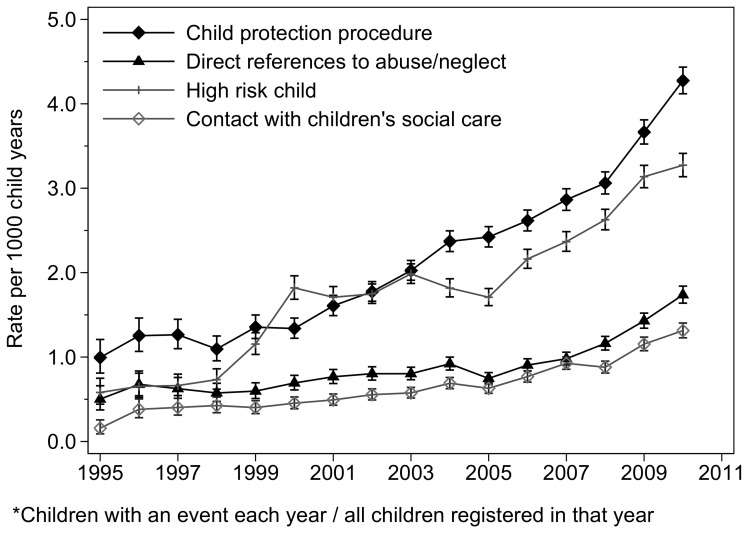
Variation of maltreatment-related codes over time, for any maltreatment-related code and each sub-category.

Overall, there was a year on year increase of 10.8% between 1995 and 2010 (95% Confidence Interval (CI) 10.5, 11.2) in the rate of any maltreatment-related codes. The annual rate of increase was similar in the four subcategories except for codes making direct references to maltreatment or out-of-home-care, where the increase was less (7.5% increase per year (95%CI 6.8, 8.3); (Figure 1 and Table 3)).

**Table 3:** Annual percentage change 1995–2010 in rate of maltreatment-related codes, by code sub-category and age.

	% change per calendar year 1995–2010 (95%CI)				
	Any maltreatment-related code	Child protection procedure	Direct reference to maltreatment or out-of-home-care	High risk child	Contact with Children's Social Care
Overall*	10.8 (10.5, 11.2)	11.6 (11.1, 12.1)	7.5 (6.8, 8.3)	11.8 (11.2, 12.4)	11.2 (10.2, 12.1)
<1 y†	13.1 (12.1, 14.0)	11.2 (9.9, 12.5)	12.1 (10.0, 14.3)	16.1 (14.4, 17.8)	13.1 (12.1, 14.0)
1–4 y†	12.8 (12.1, 13.5)	11.7 (10.8, 12.7)	9.2 (7.5, 10.8)	18.0 (16.7, 19.4)	12.8 (12.1, 13.5)
5–9 y†	11.4 (10.7, 12.2)	12.0 (11.0, 13.0)	6.7 (5.2, 8.3)	14.3 (12.9, 15.8)	11.4 (10.7, 12.2)
10–15 y†	8.7 (8.0, 9.3)	10.7 (9.7, 11.8)	4.2 (2.9, 5.5)	7.9 (6.7, 9.1)	8.7 (8.0, 9.3)
16–17 y†	4.8 (3.7, 5.9)	11.2 (8.3, 14.1)	8.8 (5.8, 11.8)	1.2 (−0.0, 2.6)	4.8 (3.7, 5.9)

* Adjusted for sex, deprivation quintile, age and overdispersion at the practice level.

† Adjusted for sex, deprivation quintile and overdispersion at the practice level.

The increase was steepest for children under five years old (Table 3). The interaction between age group and deprivation quintile showed a steeper year on year increase for deprived children in the mid age ranges (1–4 y and 5–10 y) than for deprived children at the extremes of age (data not shown).

In 2010 the annual incidence of children with any maltreatment-related code was 9.5 (95%CI 9.3, 9.8) per 1000 child years, equivalent to a new code for 0.8% (95%CI 0.8, 0.8) of all children registered for any time in 2010. The incidence was highest for child protection procedures (4.3 per 1000 child years (95%CI 4.1, 4.4)) and lowest for contact with social care (1.3 per 1000 child years (95%CI 1.2, 1.4), equivalent to 0.4% (95%CI 0.4, 0.4) and 0.1% (95% CI 0.1, 0.1) of all registered children, respectively. The incidence of children with codes for child protection plans (excluding other child protection procedures) was 2.3 per 1000 child years at risk in 2010 (95%CI 2.2, 2.4), equivalent to a new code for 0.2% of all children registered in that year (95%CI 0.2, 0.2). Table 4 reports annual incidence data. Prevalence in 2010 is not shown in any tables.

**Table 4:** Rate and rate ratio of any maltreatment-related code per 1000 child years at risk (95%CI) in 2010, by child characteristic.

	Any maltreatment-related code		Child protection procedures		References to maltreatment or out-of-home care		High risk child		Contact with Children's Social Care	
	Rate*	Rate ratio**	Rate*	Rate ratio**	Rate*	Rate ratio**	Rate*	Rate ratio**	Rate*	Rate ratio**
**All**	9.5 (9.3, 9.8)	—	4.3 (4.1, 4.4)	—	1.7 (1.6, 1.8)	—	3.3 (3.1, 3.4)	—	1.3 (1.2, 1.4)	—
Boy	9.2 (8.9, 9.5)	baseline	4.2 (4.0, 4.5)	baseline	1.8 (1.6, 1.9)	baseline	3.0 (2.9, 3.2)	baseline	1.3 (1.2, 1.4)	baseline
Girl	9.9 (9.5, 10.2)	1.1 (1.0, 1.2)	4.3 (4.1, 4.6)	1.0 (0.9, 1.1)	1.7 (1.6, 1.9)	1.0 (0.9, 1.2)	3.5 (3.3, 3.8)	1.2 (1.1, 1.3)	1.3 (1.2, 1.5)	1.1 (0.9, 1.2)
<1 y♠	24.9 (23.3, 26.6)	4.3 (3.8, 4.9)	10.8 (9.8, 11.9)	8.9 (7.0, 11.4)	4.7 (4.0, 5.4)	4.0 (3.0, 5.2)	9.6 (8.6, 10.6)	3.7 (3.0, 4.6)	2.4 (2.0, 3.0)	2.2 (1.6, 3.0)
1–4 y	13.1 (12.6, 13.7)	2.4 (2.1, 2.7)	6.0 (5.6, 6.4)	5.0 (4.0, 6.3)	2.5 (2.3, 2.8)	1.9 (1.5, 2.5)	4.8 (4.5, 5.2)	2.1 (1.7, 2.5)	1.4 (1.2, 1.6)	1.3 (1.0, 1.8)
5–9 y	8.4 (8.0, 8.9)	1.6 (1.5, 1.9)	4.2 (3.9, 4.6)	3.7 (3.0, 4.7)	1.3 (1.2, 1.5)	1.1 (0.9, 1.6)	2.7 (2.4, 2.9)	1.2 (1.0, 1.5)	1.2 (1.0, 1.4)	1.2 (0.9, 1.5)
10–15 y	7.2 (6.8, 7.5)	1.4 (1.3, 1.6)	3.3 (3.1, 3.5)	2.9 (2.3, 3.7)	1.3 (1.2, 1.5)	1.2 (0.9, 1.6)	2.1 (1.9, 2.3)	0.9 (0.8, 1.1)	1.3 (1.1, 1.4)	1.3 (1.0, 1.6)
16–17 y	5.1 (4.6, 5.6)	baseline	1.1 (0.9, 1.3)	baseline	1.1 (0.9, 1.3)	baseline	2.2 (1.9, 2.6)	baseline	1.0 (0.8, 1.2)	baseline
Deprivation										
Least†	4.0 (3.7, 4.3)	baseline	4.0 (3.7, 4.3)	baseline	1.5 (1.3, 1.6)	baseline	1.7 (1.5, 1.9)	baseline	0.7 (0.5, 0.8)	baseline
2	4.6 (4.2, 4.9)	1.2 (1.0, 1.4)	4.6 (4.2, 4.9)	1.0 (0.8, 1.26)	1.6 (1.4, 1.8)	1.3 (1.0, 1.7)	1.6 (1.4, 1.9)	1.1 (0.9, 1.4)	0.6 (0.5, 0.8)	0.8 (0.6, 1.1)
3	9.1 (8.6, 9.6)	2.2 (2.0, 2.4)	9.1 (8.6, 9.6)	2.4 (2.0, 2.8)	3.9 (3.5, 4.2)	2.1 (1.7, 2.7)	3.1 (2.8, 3.4)	2.0 (1.6, 2.4)	1.3 (1.1, 1.5)	1.6 (1.2, 2.1)
4	14.2 (13.5, 14.9)	3.0 (2.7, 3.4)	14.1 (13.5, 14.9)	3.9 (3.3, 4.6)	7.1 (6.6, 7.6)	2.2 (1.7, 2.9)	4.7 (4.3, 5.1)	2.7 (2.3, 3.2)	1.8 (1.6, 2.1)	2.1 (1.2, 2.1)
Most	19.1 (18.2, 20.0)	4.5 (4.0, 5.1)	21.3 (20.4, 22.3)	5.6 (4.7, 6.6)	10.3 (9.6, 11.0)	3.9 (3.0, 5.0)	7.0 (6.4, 7.5)	4.0 (3.3, 4.8)	2.7 (2.4, 3.1)	2.7 (2.0, 3.5)
Missing	12.1 (10.5, 13.8)	2.4 (2.1, 2.9)	12.1 (10.5, 13.8)	2.4 (1.8, 3.1)	4.4 (3.5, 4.5)	2.5 (1.8, 3.7)	3.6 (2.8, 4.6)	1.8 (1.3, 2.4)	2.2 (1.6, 3.0)	2.6 (1.8, 3.8)

♠ Age at first maltreatment-related code in 2010.

† Deprivation quintiles based on Townsend score.

* Unadjusted.

** Adjusted for other characteristics in table and overdispersion.

The incidence rate of any maltreatment-related code for infants in 2010 was 24.9 per 1000 child years (95%CI 23.3, 26.6). This was just over four times higher than for children in the oldest age group (Table 4). The prevalence of any maltreatment-related code in the same year was 1.5% (95%CI 1.4, 1.6) for infants and 1.2% (95%CI (1.1, 1.2) for children aged 1–4 years. The most deprived children had an annual incidence rate of 19.1 per 1000 child years in 2010 (95%CI 18.2, 20.0), which was four and a half times higher than for the least deprived children (Table 4). There was no difference between boys and girls.

Sensitivity analyses made no qualitative difference to the incidence rates in 2010 (Table 5).

**Table 5:** Senstivity analyses.

Incidence rates per 1000 child years at risk in 2010 (95% confidence intervals)					
	Any maltreatment-related code	Child protection procedure	Direct reference to maltreatment or out-of-home-care	High risk child	Contact with Children's Social Care
Main results	9.5 (9.3, 9.8)	4.3 (4.1, 4.4)	1.7 (1.6, 1.8)	3.3 (3.1, 3.4)	1.3 (1.2, 1.4)
Including temporarily registered patients	9.6 (9.4, 9.8)	4.3 (4.1, 4.4)	1.7 (1.6, 1.8)	3.3 (3.1, 3.4)	1.3 (1.2, 1.4)
Excluding 5 m of time at risk following registration for all children, including infants	8.9 (8.7, 9.1)	4.0 (3.9, 4.2)	1.6 (1.5, 1.7)	3.0 (2.9, 3.1)	1.3 (1.2, 1.4)
Including all time at risk after registration for all children	10.6 (10.4, 10.8)	4.7 (4.5, 4.8)	2.1 (2.0, 2.2)	3.6 (3.4, 3.7)	1.4 (1.3, 1.5)

### Variation between practices

**Figure 2:**
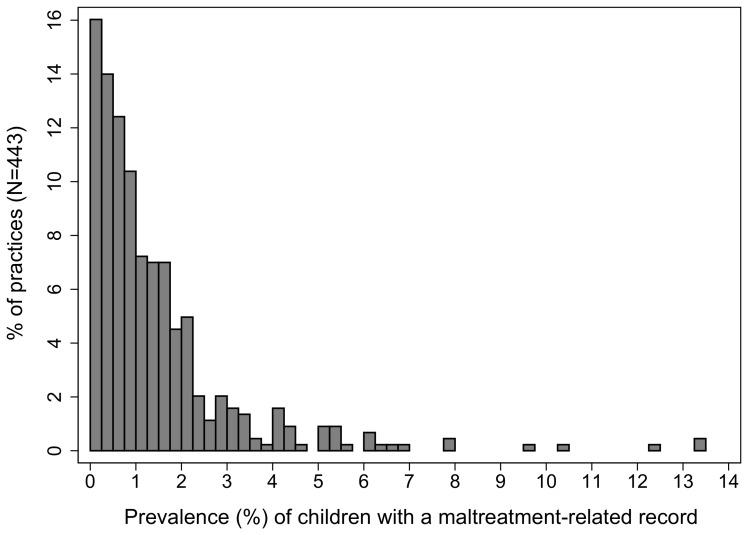
Prevalence of any maltreatment-related code from 2008 to 2010: unadjusted variation by practice.

**Figure 3:**
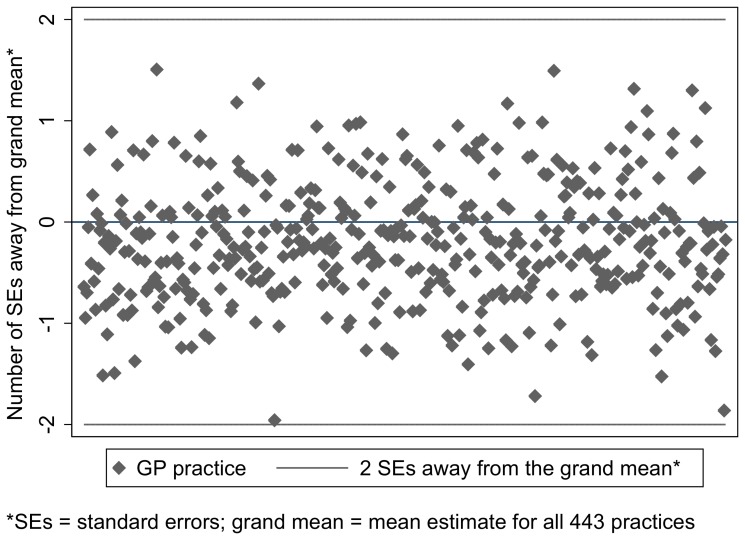
Variation between practice. Figure 3 is based on prevalence of first maltreatment-related record over three years (2008–2010). It shows the number of standard errors between practice prevalence estimates and the grand mean (mean of all 443 practices), adjusted for age, sex and deprivation.

The unadjusted prevalence of any maltreatment-related code over three years (2008–2010) ranged from zero to 13.4% with a median of 0.9% (Figure 2). Of the 433 practies, only 11 (2.5%) had no children with maltreatment-related codes in the three years. 23 (5.3%) practices had entered a relevant code for 4% or more of their registered children in the same period. After adjusting for practice case-mix (sex, age and deprivation) there was no evidence of unexplained variation beyond that due to random variation as all case-mix adjusted practice estimates were within two standard errors of the grand mean (Figure 3). There was no evidence of any effect of overdispersion on the results.

## Discussion

### Main findings

The use of maltreatment-related codes in children's primary care records has increased steeply since 1995 and is consistent across practices once case-mix and random error have been taken into account.

### Strengths and weaknesses

Our results are likely to be generalizable to UK primary care. A small validation exercise corroborated the specificity of our codes, meaning that our outcome measure is unlikely to include children without any maltreatment concerns [24].

Our results are likely to underestimate response to child maltreatment by GPs. Clinicians see coding as a complex sociotechnical issue that is part of their relationship with the patient [28]. Motivated by a desire to protect the therapeutic relationship with the family and to avoid breaching confidentiality, maltreatment-related problems are often recorded as free-text entries, scanned documents and/or in parental records - all methods of recording that were not captured by our outcome measure [10], [24]. GPs also manage families where there are known maltreatment-related concerns - but where there is no relevant information noted in any form in the child's primary care record [24]. Apart from the GP, other members of the primary health care team, such as health visitors and specially trained community nurses, may be responding to maltreatment concerns raised by patients on the GP's list, but without any notification of the problem in the primary care records. This is because in many localities, there is no shared primary care record.

Further evidence that our results underestimate the burden of maltreatment presenting to GPs comes from comparison of coded maltreatment-related problems in primary care records 2010 with community surveys (Table 6). If maltreated children present to primary care with about the same frequency as non-maltreated children [29], codes in THIN vastly underestimate the scale of the problem that is presenting to GPs. More UK evidence is needed to confirm rates of presentation by maltreated children and their families.

**Table 6:** Comparison of THIN results (2010) with evidence from other sources.

	Coded in THIN	Comparison with other data	
Measure	Freq % (95%CI*)	Freq %	Details
Maltreatment	0.8 (0.8, 0.8)	4–10	Population-based surveys of parents and/or children (self-report) [1], [2], [3].
Child Protection Plan**	0.2 (0.2, 0.2)	0.4	Children who were made the subject of a child protection plan in England 2010–11 [33]
‘High risk child’	0.3 (0.3, 0.3)	27	Children in England experiencing two or more hardships (e.g. parental depression or alcohol or substance abuse, financial stress or overcrowding; 12, 583 children and parent dyads from UK birth cohort study) [38].
Contact with children's social care	0.1 (0.1, 0.1)	5.6	Children referred to social care services for any reason in England 2010–11 [33].

* 95% confidence intervals for the annual prevalence in 2010.

** excluding other child protection procedures; the % of children with a code in THIN in 2010 was approximately half as high as the % of children who were made the subject of a Child Protection Plan consistently across all age groups.

The discrepancy between what was coded in THIN and community estimates was lowest for child protection plans and highest for ‘high risk’ child and social care referrals (Table 6). This may be because GPs are reluctant to code concerns that are below the threshold for social care child protection intervention and/or are more likely to be informed of child protection procedures than referrals to social care made by schools, the police or other healthcare professionals.

A further discrepancy compared with community studies is that younger children had higher rates of maltreatment-related codes than older children [1], [3]. This may be explained by increased GP awareness of maltreatment in younger children, by information from health visitors (who only work with preschool children), or by a lower consultation rate for older children with fewer opportunities for identification and recording [30], [31].

### Between practice-variation

Use of maltreatment-related codes was consistent across practices, once case-mix and random error were taken into account: our annual rates were not driven by a few ‘expert’ practices. However, small numbers of children with codes in each practice probably limited the power to detect moderate variation between practices.

### Explanations for the increase over time

Increasing rates of maltreatment-related codes are not explained by rising background rates of child maltreatment or related events.UK data suggest that maltreatment in the community [1], [32] and referrals to children's social care [33], [34] have been stable in recent years. There have been increases in the rate of child protection plans in the community, especially for infants, but this does not explain the rise in all four sub-categories of maltreatment-related concerns and across all age groups [33], [34].

It is unlikely that such a steady increase as seen in our results is explained by a response to a single event. There was no evidence of diagnostic transfer between codes; all four of our sub-categories increased at a broadly similar rate.

Increases may reflect system changes, such as administrators systematically coding social care correspondence and reports. However, codes reflecting judgements increased at a similar rate to those reflecting social care child protection procedures.

It seems most likely that the increasing rate of maltreatment-related codes in UK primary care is due to changes in coding behaviour and/or increased recognition by GPs. Studies have shown that QOF (pay for performance) has increased coding for a range of diseases in adults, including diseases for which coding is not incentivised under the scheme [35]. Although we found no evidence that the model with a change point in 2005 (the first full year after QOF) was a better fit for the trend over time than the log linear model, we cannot rule out the contribution of QOF to the increase in maltreatment-related codes.

### Implications

Policy-makers should consider how to build on this evidence of widespread and increasing engagement from the average GP by writing GPs into policies and systems. For example, GPs are currently isolated from other primary health colleagues by not having interoperable primary care recording systems and by the decreasing numbers of co-located health visitors. Social care could ensure that GPs are routinely informed when there are safeguarding concerns about a child, regardless of whether the child is made the subject of a child protection plan. Such information is relevant to the healthcare of the child, the parents and siblings registered with the GP.

Recording of maltreatment-related concerns is likely to increase more steeply still in light of the recent NICE and GMC guidance which recommend recording all concerns about maltreatment [4], [5]. Potentially, recording could increase from current levels (0.8% of all registered chidlren) to match the estimated incidence of maltreatment (4–10%) or of vulnerable children in the community each year (27%; Table 6). This would have major cost implications, as better recording should be accompanied by other action that benefits the child and family. The RCGP have developed a simple and feasible approach to coding maltreatment-related concerns (www.clininf.ed/maltreatment) [24].

Currently, we do not know how far GPs are identifying and responding to concerns in children whose problems are already known to other services or whether these children have other service contacts. Studies using linked primary, secondary and social care data may help identify patterns of contacts and concerns and further illuminate the role that the GP may play in relation to other services.

Recording is a necessary but not sufficient part of responding to maltreatment. Other recommended responses include discussion with colleagues, use of primary care team meetings, information gathering and sharing and, where thresholds are met, referral to social care and on-going involvement in official child protection procedures [4], [5], [36]. Little is known about the effectiveness of these recommended responses. Without effective interventions, increased coding could have net harm through sharing stigmatising information or jeopardising therapeutic relationships. Consequently, interventions to improve maltreatment-related coding need to be evaluated for their impact on subsequent action and outcomes for the child and family. This should happen as part of a wider multi-component intervention for managing child maltreatment in primary care.
